# Inhibition of Mitochondrial-Associated Protein MAGMAS Resensitizes Chemoresistant Prostate Cancer Cells to Docetaxel

**DOI:** 10.3390/cancers17091535

**Published:** 2025-04-30

**Authors:** Alfonso M. Durán, Kristen Whitley, Krystal Santiago, Christian Yoo, Giancarlo Valdez, Kai Wen Cheng, Pedro Ochoa, David de Semir, Joanne Xiu, Parthiban Chokkalingam, Sasmita Das, Eric S. Schaefer, Steven P. Rowe, Bhaskar C. Das, Carlos A. Casiano, Frankis Almaguel

**Affiliations:** 1 Center for Health Disparities and Molecular Medicine, Loma Linda University School of Medicine, Loma Linda, CA 92350, USA; aduran@llu.edu (A.M.D.); krystalsantiago@students.llu.edu (K.S.); yooknowit@gmail.com (C.Y.); kaiwencheng@llu.edu (K.W.C.); pedroochoa@llu.edu (P.O.); ccasiano@llu.edu (C.A.C.); 2 Department of Basic Sciences, Loma Linda University School of Medicine, Loma Linda, CA 92350, USA; 3 Cancer Center, Loma Linda University Health, Loma Linda, CA 92354, USA; 4 Caris Life Sciences, Phoenix, AZ 85040, USA; ddesemir@carisls.com (D.d.S.); jxiu@carisls.com (J.X.); 5 Department of Pharmaceutical Sciences, School of Pharmacy and Pharmaceutical Sciences, University at Buffalo, Buffalo, NY 14215, USA; pchokkal@buffalo.edu (P.C.); sasmitad@buffalo.edu (S.D.); bhaskard@buffalo.edu (B.C.D.); 6 Highlands Oncology, Rogers, AR 72758, USA; eschaefer@hogonc.com; 7 Department of Radiology, University of North Carolina, Chapel Hill, NC 27514, USA; steven_rowe@med.unc.edu; 8 Department of Medicine, Rheumatology Division, Loma Linda University School of Medicine, Loma Linda, CA 92350, USA

**Keywords:** chemoresistance, docetaxel, prostate cancer, MAGMAS, mitochondria

## Abstract

Background/Objectives: Metastatic prostate cancer (PCa) is the leading cause of cancer-related deaths and a major contributor to cancer mortality in men. Most patients with metastatic PCa eventually develop metastatic castration-resistant prostate cancer (mCRPC), characterized by resistance to treatment with androgen-deprivation therapy, and often later the development of resistance to other types of agents. MAGMAS, a 13.8 kDa mitochondrial-associated protein, facilitates the import of nuclear-encoded proteins into the mitochondrial matrix. Overexpression of MAGMAS has been observed in several aggressive cancers, including breast, glioblastoma, and prostate cancer. When overexpressed, MAGMAS acts as a cytoprotective protein by scavenging reactive oxygen species (ROS), maintaining ROS levels that support cell proliferation while avoiding the induction of apoptosis. This study investigates the role of MAGMAS in therapy resistance in PCa cells. Methods/Results: Quantitative immunoblotting revealed that MAGMAS is endogenously upregulated in docetaxel-resistant (DR) PCa cell lines compared to their docetaxel-sensitive parental counterparts. While MAGMAS depletion alone did not affect the survival of DR cells, it significantly sensitized them to docetaxel (DTX), as indicated by a marked reduction in clonogenic potential. Additionally, transient knockdown of MAGMAS in these resistant cells significantly decreased the levels of ABCB1 protein. Consistent with these findings, sub-therapeutic inhibition of MAGMAS using the novel BT#9 inhibitor, in combination with increasing concentrations of DTX, enhanced the sensitivity of DR cells to DTX, as demonstrated by proliferation and clonogenic assays. Lastly, RNA tumor expression predicts overall survival (OS). Conclusions: These results implicate MAGMAS in PCa chemoresistance and suggest that targeting this protein could provide a novel therapeutic strategy for treating DR tumors.

## Simple Summary

This study examined a protein called MAGMAS in prostate cancer cells that are resistant to docetaxel (a common chemotherapy drug) compared to sensitive cells. MAGMAS levels were found to be much higher in the resistant cells. However, the reduction in MAGMAS levels in these resistant cells led to a noticeable drop in another protein called ABCB1, which helps cancer cells resist drugs. Additionally, resistant cells required more docetaxel or MAGMAS blocker, BT#9, to be affected compared to sensitive cells. However, when a low dose of BT#9 was used together with docetaxel, the resistant cells became more sensitive to docetaxel, needing lower concentrations to reduce cellular growth. In short, targeting MAGMAS with BT#9 could help make therapy-resistant prostate cancer cells respond better to docetaxel, providing a possible new way to tackle resistance to treatment.

## 1. Introduction

Prostate cancer (PCa) is the most commonly diagnosed non-cutaneous malignancy and the second leading cause of cancer-related death among men in the United States [1]. At diagnosis, PCa is typically androgen-driven, relying on ligand-mediated signaling through the androgen receptor (AR) to support tumor growth and progression [2]. As a result, androgen-deprivation therapy, which inhibits AR signaling, has long been a cornerstone of initial treatment, leading to decreased PSA levels and tumor volume [3,4]. More recently, next-generation Androgen Receptor Axis-Targeted therapy (ARAT), such as abiraterone acetate and enzalutamide, has significantly improved overall survival in patients with advanced PCa [5,6,7]. However, emerging clinical evidence indicates that after prolonged AR signaling inhibition, ~15–25% of patients with advanced PCa develop tumors characterized by AR independence, neuroendocrine phenotype, and stemness features [8,9,10,11]. This phenotypic change is defined as treatment-emergent neuroendocrine prostate cancer (t-NEPC) [12,13].

The molecular mechanisms driving neuroendocrine trans-differentiation of prostate tumors remain incompletely understood. Nevertheless, comprehensive next-generation sequencing studies have identified frequent upregulation and amplification of MYC signaling pathways, particularly involving the NMYC proto-oncogene protein, in t-NEPC tumors [14]. Additionally, these tumors often exhibit loss of the tumor suppressor genes *RB1* and *TP53* [15]. Notably, in preclinical models, NMYC overexpression in human prostate epithelial cells results in the development of aggressive tumors, resembling neuroendocrine-like cells, with upregulated neuroendocrine markers and diminished AR signaling [16,17], underscoring MYC pathway activation as a critical driver of t-NEPC differentiation.

The clinical management of t-NEPC is predominantly based on cytotoxic chemotherapy, often using platinum-based regimens in combination with taxanes, such as docetaxel (DTX) and etoposide [18,19,20,21]. However, the response to these therapies is generally short-lived, with limited survival benefits. In t-NEPC preclinical models, MYC overexpression is associated with metabolic reprogramming, characterized by a shift toward oxidative phosphorylation (OXPHOS), particularly in DTX-resistant tumor cells [22]. Interestingly, MYC activation in these models is linked to an increase in mitochondrial DNA (mtDNA), with the highest levels observed in advanced drug-resistant PCa tumors [23]. Although the role of mitochondrial metabolic alterations in t-NEPC drug resistance remains underexplored [24], it is well-established that mitochondrial adaptations contribute to drug resistance in cancer, including PCa [25,26,27]. Recent studies have specifically highlighted the importance of mitochondrial dysfunction and alterations in taxane resistance [28,29].

The objective of the present study was to investigate whether targeting the mitochondria-associated protein MAGMAS (Granulocyte–Macrophage Colony-Stimulating Factor Signaling Molecule) could restore DTX sensitivity in chemoresistant t-NEPC-like cells. MAGMAS has been implicated in protein transport and mitochondrial biogenesis [30], and its overexpression is associated with resistance to oxidative stress through the upregulation of antioxidant enzymes in PCa models [31,32]. MAGMAS has also been reported to be overexpressed in aggressive tumors, including prostate, ovarian, and central nervous system tumors [33,34,35], suggesting that MAGMAS overexpression may play a significant role in acquired treatment resistance in these cancers. Therefore, we sought to elucidate the role of MAGMAS in mediating taxane resistance in t-NEPC-like cells.

## 2. Materials and Methods

### 2.1. Cell Lines and Chemicals

PCa cell lines PC3, DU145, and 22Rv1 were acquired from the American Type Culture Collection (Manassas, VA, USA, Cat# ATCC-CRL-1435, ATCC-HTB-81, and ATCC-CRL-2505, respectively) and cultured in supplemented RPMI-1640 medium (Gen Clone^®^, Genesee Scientific, El Cajon, CA, USA, Ref# 25-506) [36]. Taxane-resistant cell lines were developed as previously described [37,38]. MAGMAS inhibitor BT#9 was synthesized in Dr. Das’s laboratory using previously established procedures [39]. PC3, DU145, and 22Rv1 were authenticated as previously reported [36].

### 2.2. Antibodies

Rabbit antibody targeting MAGMAS (Cat# 15321-1-AP) protein was acquired from Proteintech^®^ (Rosemont, IL, USA). Rabbit monoclonal antibody targeting ABCB1 (Cat# 13342) protein and mouse monoclonal antibody targeting beta-actin (Cat# 3700) were acquired from Cell Signaling Technology (Danvers, MA, USA).

### 2.3. Immunoblotting

Cell lysates were prepared as described previously [37,38], to ensure equal loading of proteins separated on individual lanes by Mini-PROTEAN^®^ TGX™ Precast Protein 4–15% (Cat# 4561084, Hercules, CA, USA). Electrophoresis was followed by protein transfer to Bio-Rad Immun-Blot^®^ Low Fluorescence PVDF membranes (Cat# 1620262). Membranes were blocked with Bio-Rad EveryBlot blocking buffer (Cat# 12010020). Membranes were incubated with secondary anti-rabbit IgG StarBright™ Blue 700 (Bio-Rad, Contra Costa, CA, USA, Cat# 12004161), anti-mouse IgG StarBright™ Blue 700 (Bio-Rad, Cat# 12004158), or anti-mouse IgG (H + L) DyLight™ 488 (Invitrogen, Carlsbad, CA, USA, Cat#35503). Immunoreactive protein bands were detected by the Bio-Rad ChemiDoc MP Imaging System. Protein bands from at least three independent blots were scanned and quantified using Bio-Rad Image Lab software (version 6.1.0, build 7). Signal intensities were normalized to β-actin loading controls to calculate relative fold abundance.

### 2.4. Quantitative Real-Time PCR

Quantitative real-time PCR (qPCR) was performed as described previously [40]. MAGMAS primer sequences were designed using Primer3 software and synthesized by Integrated DNA Technologies (IDT). β-actin mRNA served as the internal control, and data were normalized to corresponding control values.

### 2.5. MTT Viability Assay and Determination of IC_50_ Values

Cells were seeded in 96-well plates as previously described [36] and treated with increasing concentrations of DTX (0–1000 nM) or BT#9 for up to 72 h. Treatments were conducted in at least three independent experiments, each with three biological replicates. Following treatment, cell viability was assessed using a 3-(4,5-dimethylthiazol-2-yl)-2,5-diphenyltetrazolium bromide (MTT) assay, as previously reported [36].

### 2.6. RNA Interference

PC3-DR and DU145-DR cells (50,000 cells per well) were cultured on 6-well plates and transfected 24 h later with either scrambled (SD) negative control (Integrated DNA Technologies, Coralville, IA, USA, Cat# 51-01-19-09) or 1 nM MAGMAS DsiRNAs for up to 96 h. The duplex siRNA sequences used were as follows: si-MAGMAS (5′-GAACACUUAUUUAAGGUGAAUGATA-3′) and (3′-UACUUGUGAAUAAAUUCCACUUACUAU-5′). siRNA transfections were performed as described [36,41] and confirmed by immunoblotting.

### 2.7. Clonogenic Assays

PC3-DR and DU145-DR cells were transfected with individual siRNAs and grown in RPMI-1640 medium supplemented for 72 h. We previously described the clonogenic assay protocol in detail [36]. Images of the stained colonies were acquired using the Bio-Rad ChemiDoc MP Imaging System, and quantification was performed using the automated colony counting capability of Image J software version 1.54h following identical parameters for each well [42].

### 2.8. Kaplan–Meier Curve Data

Real-world clinical data were obtained from insurance claims. Real-world overall survival (OS) was defined as the period from tissue collection to the date of the patient’s last known clinical activity. Kaplan–Meier survival estimates were generated for cohorts defined by molecular characteristics and treatments. Hazard ratios (HRs) were computed utilizing the Cox proportional hazards model, and significant differences in survival times were assessed with the log-rank test, where *p* < 0.05 was considered significant.

## 3. Results

### 3.1. MAGMAS Is Endogenously Overexpressed in DTX-Resistant PCa Cells

**Figure 1:**
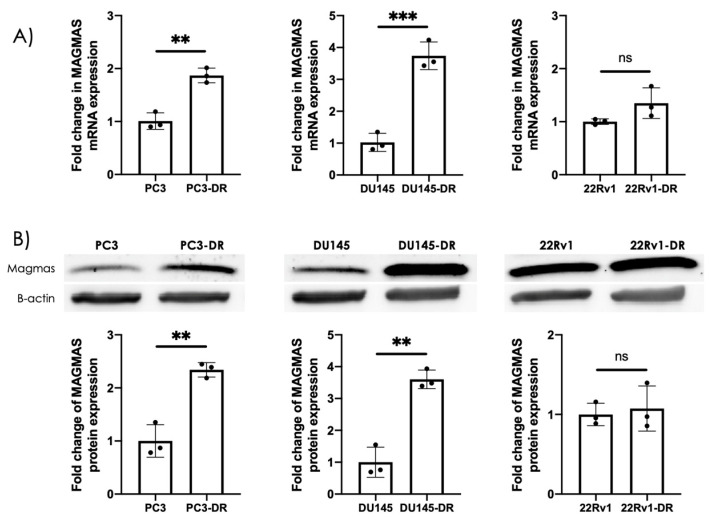
MAGMAS is overexpressed in DTX-resistant DU145 and PC3 cells. (**A**) MAGMAS mRNA levels were quantified by qPCR using RNA isolated from PC3, PC3-DR, DU145, DU145-DR, 22Rv1, and 22Rv1-DR cells across at least three independent experiments. Statistical significance was assessed relative to parental control cells using Student’s *t*-test (** *p* < 0.01, *** *p* < 0.001). (**B**) Top panels: Representative immunoblots showing MAGMAS protein expression in lysates from the same cell lines, detected using a rabbit anti-MAGMAS antibody specific for this ~14 kDa protein. Bottom panels: Bar graphs showing quantification of fold change in protein expression from at least three independent experiments per cell line via Bio-Rad Image Lab analysis, with values normalized to β-actin. Statistical significance was determined in comparison to control parental cells using Student’s *t*-test (** *p* < 0.01). Error bars represent mean ± standard deviation (SD). (File S1: The original version the Western blot image in Figure 1B).

The PC3 and DU145 cell lines have been shown to express neuroendocrine markers [23,43]. In addition, their DTX-resistant counterparts upregulate c-MYC, neuroendocrine markers, and stemness markers [22,37] (Supplementary Materials Figure S1). Because of these features, these cell lines are considered NEPC-like [44,45,46]. We determined the expression of MAGMAS in the DTX-resistant AR-independent cell lines DU145-DR and PC3-DR, compared to the drug-sensitive parental DU145 and PC3 cells. MAGMAS expression was significantly upregulated at both the mRNA and protein levels in the chemoresistant PC3-DR and DU145-DR cell lines (Figure 1A,B), while no significant change was observed in the AR-dependent 22Rv1-DR cells. Specifically, MAGMAS transcript levels increased 1.87-fold in PC3-DR and 3.74-fold in DU145-DR cells compared to their respective parental lines (Figure 1A, left panel). To determine whether this transcriptional upregulation was reflected at the protein level, total cell lysates from PC3, PC3-DR, DU145, DU145-DR, 22Rv1, and 22Rv1-DR were analyzed by immunoblotting using a MAGMAS-specific antibody. Consistent with the mRNA data, MAGMAS protein expression was markedly increased in PC3-DR and DU145-DR cells but remained unchanged in 22Rv1-DR cells relative to their parental counterparts (Figure 1B).

### 3.2. MAGMAS Depletion Leads to Decreased ABCB1 Abundance in DTX-Resistant PCa Cells

**Figure 2:**
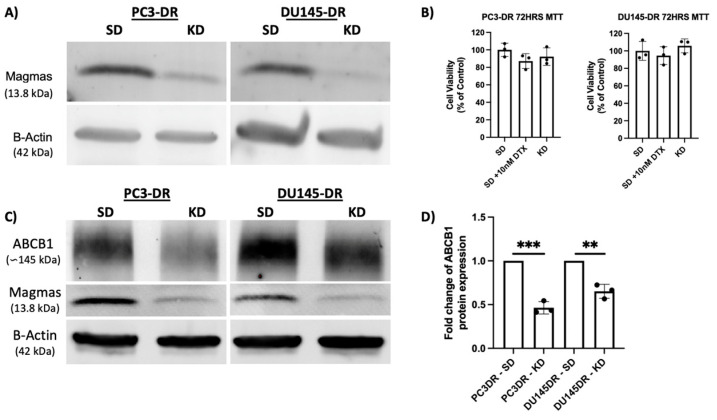
Transient knockdown of MAGMAS in DU145-DR and PC3-DR cells. (**A**) MAGMAS knockdown (KD) was confirmed by immunoblotting using a rabbit anti-MAGMAS antibody in DU145-DR and PC3-DR cells transfected with DsiRNA MAGMAS as compared to cells transfected with the SD negative control. (**B**) MTT assay bar graph demonstrating percentage of PC3-DR and DU145-DR cell viability in scramble duplex control (SD), SD + 10 nM DTX, and MAGMAS KD alone. (**C**) Immunoblots showing that transient depletion (72h) of MAGMAS in PC3-DR and DU145-DR cells attenuates ABCB1 protein levels. (**D**) Bar graphs showing quantification of fold change in ABCB1 levels after MAGMAS KD from at least three independent experiments per cell line via Bio-Rad Image Lab analysis, with values normalized to β-actin. Statistical significance was determined in comparison to SD controls using Student’s *t*-test (** *p* < 0.01, *** *p* < 0.001). Error bars represent mean ± standard deviation. (File S2: The original version the Western blot image in Figure 2A; File S3: The original version the Western blot image in Figure 2C).

After establishing the upregulation of MAGMAS in PC3-DR and DU145-DR cells, we sought to determine if using specific siRNAs targeting MAGMAS impacted cell viability. For these experiments, we transiently knocked down MAGMAS in our DTX-resistant cellular models (Figure 2A) and determined if MAGMAS knockdown alone decreased the viability of DTX-resistant cells. Interestingly, we did not observe any differences in cell viability when comparing scramble negative control, maintenance DTX dose (10 nm) plus siRNA, and siRNA alone for PC3-DR and DU145-DR cells (Figure 2B).

We confirmed the DTX-resistant status of PC3-DR and DU145-DR cell lines by validating upregulation of ATP-binding cassette sub-family B member 1 (ABCB1), also known as multidrug resistance 1 (MDR1), compared to the sensitive cells (Supplementary Data Figure S1). Next, we sought to determine if depletion of MAGMAS impacted the levels of ABCB1 protein. We found that transient knockdown of MAGMAS in PC3-DR and DU145-DR cells significantly decreased ABCB1 protein levels (Figure 2C,D). Specifically, PC3-DR cells displayed a 35% reduction in ABCB1 protein levels compared to SD control (Figure 2D). Similarly, DU145-DR cells displayed a 54% reduction in ABCB1 protein levels compared to SD control (Figure 2D).

### 3.3. MAGMAS Depletion Inhibits the Clonogenicity Capacity of DTX-Resistant PCa Cells

**Figure 3:**
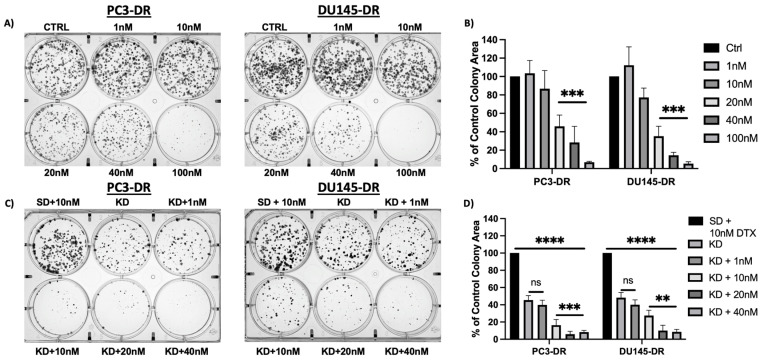
Knockdown of MAGMAS resensitizes PC3-DR and DU145-DR cells to DTX, shown by a decrease in clonogenicity. (**A**) Representative images of clonogenic assay plates show decreased colony formation in PC3-DR and DU145-DR cells exposed to DTX concentrations above 10 nM. (**C**) Representative images of clonogenic assay plates show decreased colony formation in PC3-DR and DU145-DR cells with MAGMAS KD compared to SD control cells in the presence and absence of DTX. Colonies were counted after 10 days of treatment. (**B**,**D**) Adjacent bar graphs show quantification of PC3-DR and DU14-DR colonies and represent the average of colonies counted in at least three independent experiments. Error bars represent mean ± standard deviation. Statistical significance was determined by comparing the values to control and cells transfected with SD control + 10 nM DTX with values for cells with MAGMAS knockdown in the presence or absence of DTX, using one-way ANOVA, followed by post hoc pairwise analysis with Bonferroni correction (** *p* < 0.01, *** *p* < 0.001, **** *p* < 0.0001).

The upregulation of MAGMAS is associated with cellular protection and survival [31,32]. To evaluate the role of MAGMAS in chemoresistant cell survival, we examined the impact of MAGMAS silencing on colony formation in PC3-DR and DU145-DR cells. Knockdown of MAGMAS significantly reduced clonogenic capacity in both cell lines (Figure 3D). Furthermore, exposure to increasing concentrations of DTX led to a dose-dependent reduction in colony formation in both control and MAGMAS-depleted cells, with the greatest suppression observed in the combination treatment group (Figure 3B,D). We did not include parental sensitive cells in this analysis because they display lower clonogenic capacity compared to DR cells and do not form clones in the presence of DTX. 

### 3.4. DTX-Resistant PCa Cells Exhibit Resistance to MAGMAS Inhibitor BT#9

**Figure 4:**
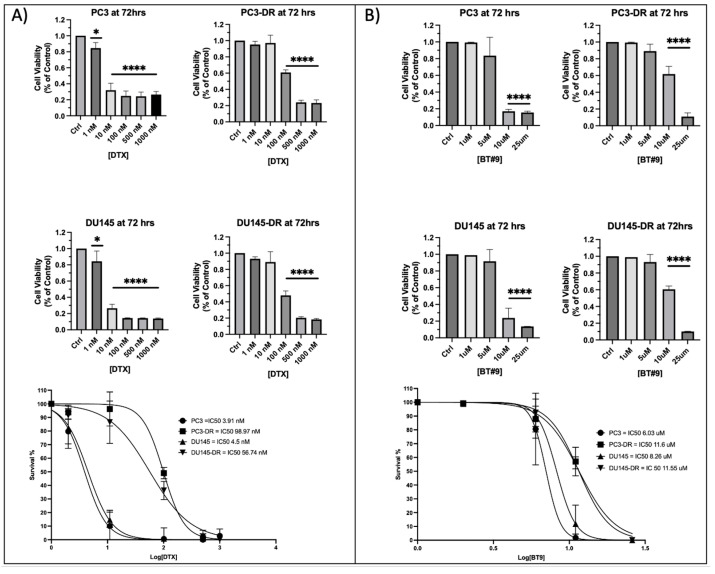
PC3-DR and DU145-DR cells are resistant to MAGMAS inhibitor BT#9. (**A**,**B**) Bar graphs showing cell viability of PC3, PC3-DR, DU145, and DU145-DR cells following 72 h treatment with increasing concentrations of DTX (1 nM, 10 nM, 100 nM, 500 nM, 1000 nM) or BT#9 (1 µM, 5 µM, 10 µM, 25 µM), as measured by MTT assay. Data represent the mean of at least three independent experiments performed in triplicate, normalized to untreated controls. Error bars indicate mean ± standard deviation. Statistical significance was assessed by one-way ANOVA with Bonferroni post hoc correction (* *p* < 0.05, **** *p* < 0.0001). (**A**,**B**) Bottom panels represent best-fit response curves used to calculate IC_50s_ for DTX and BT#9 treatments.

To validate the DTX-resistance status of PC3-DR and DU145-DR cells, we treated these chemoresistant lines and their respective parental, drug-sensitive counterparts with increasing concentrations of DTX for up to 72 h (Figure 4A, top panels) and determined the approximate half-maximal inhibitory concentration (IC_50_). As expected, PC3-DR and DU145-DR cells exhibited significantly higher IC_50_ values (approximately 100 nM and 57 nM, respectively) compared to their parental PC3 and DU145 cells (IC_50_ ≈ 3.9 nM and 4.5 nM, respectively). Best-fit dose–response curves demonstrated a statistically significant increase in cell survival across all tested concentrations (10–100 nM) in the resistant lines relative to their parental counterparts (*p* < 0.0001; Figure 4A, bottom panels). We confirmed previous reports demonstrating that the docetaxel-sensitive PC3 and DU145 cell lines respond to treatment with 10 μM of the established MAGMAS inhibitor BT#9 [32]. As anticipated, the DTX-resistant variants exhibited higher IC_50_ values compared to their respective parental cell lines (Figure 4B, *p* < 0.0001).

### 3.5. Sub-Therapeutic Inhibition of MAGMAS with BT#9 Sensitizes Chemoresistant PCa Cells to Docetaxel

**Figure 5:**
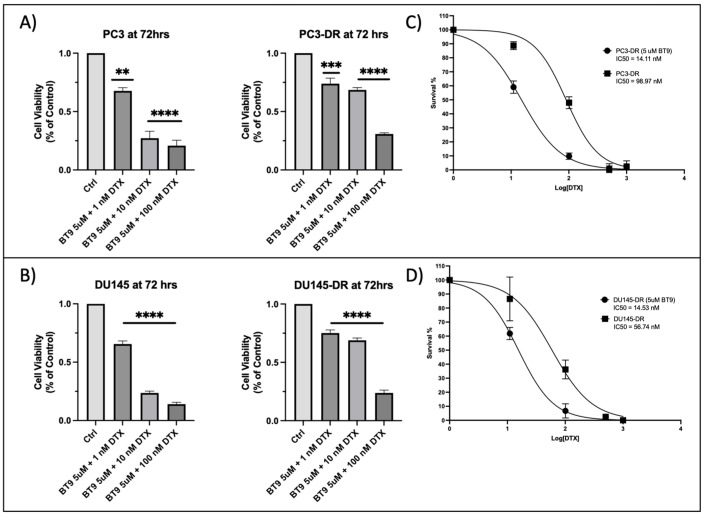
Sub-therapeutic inhibition of MAGMAS sensitizes PC3-DR and DU145-DR cells to DTX. (**A**) MTT assay bar graphs demonstrating PC3 and PC3-DR percentage of cell viability vs. concentration of 5 uM BT#9 + DTX (1 nM, 10 nM, 100 nM), control cells treated with 5 uM BT#9 alone. Adjacent to bar graphs, we show corresponding IC_50_ curves. (**B**) MTT assay bar graphs demonstrating DU145 and DU145-DR percentage of cell viability after cotreatment with BT#9 (5 uM) and DTX (1 nM, 10 nM, 100 nM), control cells treated with 5 uM BT#9 alone. Statistical significance was determined by comparing the values to control and cells treated with 5 uM BT#9 with increasing concentrations of DTX (1 nM, 10 nM, and 100 nM) using one-way ANOVA, followed by post hoc pairwise analysis with Bonferroni correction (** *p* < 0.01, *** *p* < 0.001, **** *p* < 0.0001). (**C**,**D**) represent best-fit response curves used to calculate IC_50s_ of DTX dose response alone vs. cotreatment with sub-therapeutic 5 uM BT#9 in the presence of increasing doses of DTX. Each graph represents the average of at least three independent experiments in triplicates normalized to untreated controls. Error bars represent mean ± standard deviation.

Given the profound resensitization of DR cells to DTX after depletion of MAGMAS by siRNA, it was necessary to understand if the MAGMAS inhibitor BT#9, described previously [32,39], demonstrates similar observations. For these experiments, we treated PC-3, PC3-DR, DU145, and DU145-DR cells with combination treatments of DTX (1 nM, 10 nM, and 100 nM) and 5 uM BT#9 for up to 72 h and determined the approximate IC_50_ (Figure 5A,B). We treated the cells with 5 uM BT#9 to determine if a non-cytotoxic dose (see bar graphs in Figure 4A,B) was sufficient to sensitize cells to DTX. Next, we compared the IC_50s_ of DTX alone and cotreatment with a non-cytotoxic concentration of BT#9 (5 uM) plus DTX in the PC3-DR and DU145-DR cell lines (Figure 5C,D). We found that cotreatment with sub-therapeutic BT#9 plus DTX sensitized the chemoresistant cells to DTX, decreasing the IC_50_ for PC3-DR by 90% (IC_50_ PC3-DR DTX alone = 98.97 vs. IC_50_ PC3-DR BT#9 + DTX = 11.71, Figure 5C). Similarly, cotreatment with BT#9 plus DTX sensitized DU145-DR cells to DTX, decreasing the IC_50_ by ~80% (IC_50_ DU145-DR DTX alone = 56.74 vs. IC_50_ DU145-DR BT#9 + DTX = 12.85, Figure 5D).

**Figure 6:**
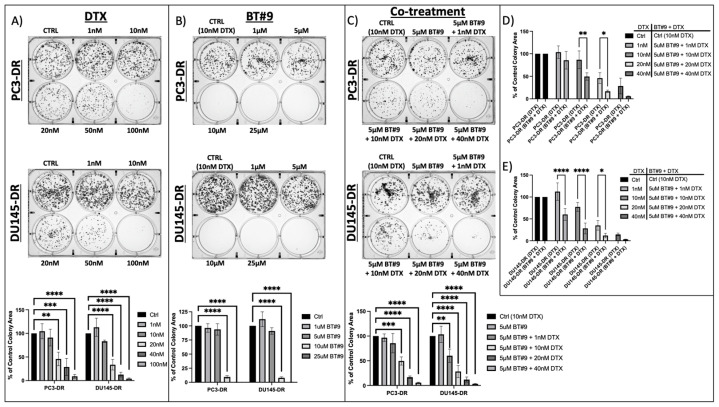
Sub-therapeutic inhibition of MAGMAS decreases clonogenic potential in PC3-DR and DU145-DR cells. (**A**,**B**) Representative images of colony formation assay plates showing decreased clonogenicity in PC3-DR and DU145-DR cells in the presence of DTX or BT#9 alone compared to controls (untreated cells and 10 nM DTX, respectively). Colonies were counted after 10 days of treatment. Bar graphs show quantification of PC3-DR and DU145-DR colony area coverage from at least three independent experiments. Standard deviation was calculated. (**C**) Representative images of colony formation assay plates showing a decrease in clonogenicity in PC3-DR and DU145-DR cells in the presence of a non-inhibitory colony formation concentration of BT#9 (5 uM) + increasing concentrations of DTX (1 nM, 10 nM, 20 nM, and 40 nM) compared to control (10 nM DTX). Bar graphs show quantification of PC3-DR and DU145-DR colony area coverage from at least three independent experiments. Standard deviation was calculated. (**D**,**E**) Bar graphs show colony quantifications comparing DTX alone vs. combination treatment of BT#9 + DTX. Statistical significance was determined by one-way ANOVA or two-way ANOVA, followed by post hoc pairwise analysis with Bonferroni correction (* *p* < 0.05, ** *p* < 0.01, *** *p* < 0.001, **** *p* < 0.0001).

Next, we assessed the clonogenic potential of PC3-DR and DU145-DR cells after treatment with DTX and BT#9, alone or in combination. The results demonstrated that treatment with DTX alone significantly reduced the clonogenicity of PC3-DR and DU145-DR at 20 nM DTX, *p* < 0.01 and *p* < 0.0001, respectively (Figure 6A). Additionally, we observed that treatment with BT#9 alone only significantly decreased clonogenic potential at 10 uM in the DR cells. As expected, the combination of sub-therapeutic MAGMAS inhibition by BT#9 (5 uM), which did not inhibit colony formation (Figure 6B), with 10 nM DTX in PC3-DR cells significantly reduced colony formation by 37% (*p* < 0.01) compared to control cells (Figure 6C,D). Similarly, treatment with 5 uM BT#9 in combination with 1 nM DTX in DU145-DR cells significantly reduced colony formation by approximately 40% (*p* < 0.0001) compared to control cells (Figure 6E). These findings indicated that the combination of a non-cytotoxic BT#9 concentration (5 uM) and low-dose DTX sensitized the chemoresistant cells to DTX compared to the controls.

### 3.6. Decreased OS in PCa Patients According to PAM16 RNA Tumor Expression

**Figure 7:**
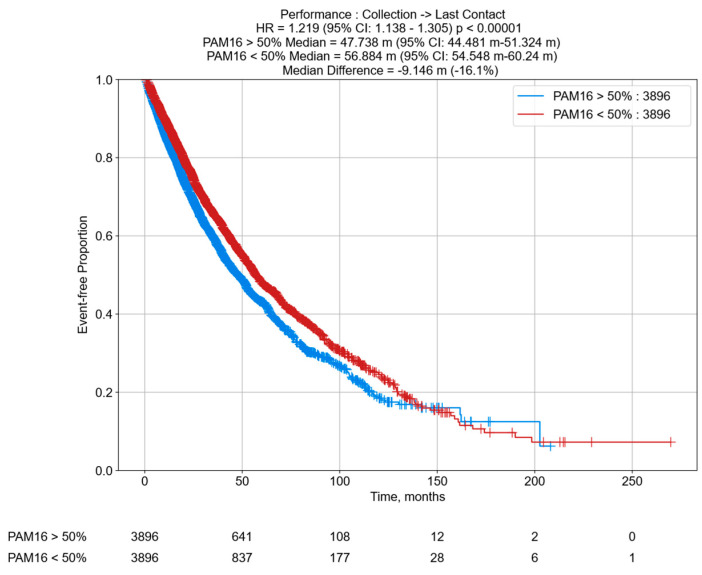
Overall survival (OS) of PCa cohort. Kaplan–Meier curves depicting OS for PCa tumors expressing high levels of PAM16 > 50th percentile (*n* = 3896) vs. PCa tumors expressing low levels of PAM16 < 50th percentile (*n* = 3896). OS was calculated from time of tissue collection to last contact.

To explore the prognostic associations of MAGMAS, gene alias PAM16, and RNA levels in PCa patients, we analyzed the OS of patients in the CARIS Life Sciences clinico-genomic database. Among the PCa patient cohort (*n* = 7792), all samples with high PAM16 expression (PCa PAM16 > 50th) from patients who received any clinically relevant anti-androgen therapy or chemotherapy at any point during treatment exhibited a shorter median OS (−9.146 months; HR = 1.219; 95% CI: 1.138–1.305; *p* < 0.00001) compared to patients with low PAM16 expression (PCa PAM16 < 50th) (Figure 7). These findings highlight the potential translational utility of MAGMAS expression as a predictive biomarker for OS outcomes in PCa patients.

## 4. Discussion

The medical management of advanced PCa, particularly with ADT and ARAT, has significantly improved patient survival, as shown in the STAMPEDE and LATITUDE trials [47,48]. However, prolonged interruption of the androgen-signaling axis can induce lineage plasticity. This process allows AR-dependent adenocarcinoma cells to transition to AR-independent phenotypes, such as t-NEPC [49,50]. This phenotypic shift is often associated with poor prognosis and heightened resistance to therapy [51,52]. While the precise mechanism underlying the development of AR independence remains unclear, MYC signaling has been shown to play a central role in t-NEPC progression [14,15].

One key consequence of MYC activation is the enhancement mitochondrial biogenesis [23,53,54]. Previous work from our group has demonstrated significant upregulation of MYC signaling and cancer stem cell markers in neuroendocrine-like PCa models resistant to therapy [37]. Furthermore, MYC is part of the glucocorticoid receptor-lens epithelium-derived growth factor p75 (GR-LEDGF/p75) transcriptional network, which contributes to drug resistance in PCa cells through regulation of multiple pathways, including oxidative stress and apoptosis [36,55].

In the present study, we identify MAGMAS as being highly overexpressed in MYC-driven DR cells. The role of mitochondria in therapy resistance is well-documented [26,56,57]. Notably, MAGMAS overexpression is linked to reduced reactive oxygen species (ROS) levels, which helps prevent ROS-mediated activation of caspases 3/7—key mediators of apoptosis [31]. Previous studies have suggested that MAGMAS reduces reactive oxygen species (ROS) by enhancing electron transport chain (ETC) activity and upregulating key antioxidant enzymes, including magnesium-dependent superoxide dismutase and glutathione peroxidase, thereby increasing the cell’s overall antioxidant capacity [31]. Consistent with these findings, MAGMAS is overexpressed in multiple tumor types and has been shown to be responsive to chemotherapy exposure [33,34,58].

Interestingly, genetic silencing of MAGMAS did not affect baseline cell viability; however, its depletion significantly enhanced the sensitivity of chemoresistant PCa cells to DTX. We also provide preliminary evidence suggesting that MAGMAS depletion may disrupt ABCB1 abundance. This is significant because ABCB1 plays a central role in the efflux of DTX from cancer cells, and ABC transporters are critical mechanisms by which chemoresistant cells evade drug accumulation [59,60]. Interestingly, ABC transporters are reported to use mitochondrial-derived ATP to power drug efflux from cancer cells [61]. In line with this, MAGMAS inhibition has been shown to reduce oxygen consumption rates (OCRs) in a dose-dependent manner [58], suggesting a link between MAGMAS activity and mitochondrial function.

Furthermore, we demonstrated that sub-therapeutic inhibition of MAGMAS using the novel inhibitor BT#9 reverses DR in chemoresistant cells, as evidenced by a significant reduction in IC_50_ values and clonogenic potential. Mitochondrial inhibitors hold promise in PCa treatment by targeting the metabolic vulnerabilities of cancer cells, particularly their dependence on oxidative phosphorylation (OXPHOS) for energy production. In advanced PCa, cells often shift their metabolism toward OXPHOS, making them more susceptible to mitochondrial-targeted therapies [62,63]. Agents such as metformin and phenformin have shown potential in disrupting mitochondrial function and reducing tumor growth in preclinical models [64]. These inhibitors may also enhance the efficacy of standard treatments, such as ADT, by impairing the cancer cells’ metabolic compensation mechanisms [65]. Another potential advantage is the selective targeting of cancer cells with high mitochondrial dependence, reducing the proliferation of more aggressive, androgen-independent tumor subtypes.

However, targeting mitochondria in PCa presents challenges. One major issue is the metabolic heterogeneity of this malignancy, as different stages and subtypes vary widely in mitochondrial function and energy requirements [63]. This variability complicates the ability to predict which patients will respond to mitochondrial-targeted therapies. Our findings suggest that MAGMAS could serve as a biomarker to identify patients more likely to benefit from such treatments. A further concern is that cancer cells may bypass mitochondrial inhibition by switching to glycolysis, a phenomenon known as metabolic reprogramming, potentially diminishing the effectiveness of mitochondrial inhibitors [66]. Our study, however, offers a promising approach by demonstrating that sub-therapeutic concentrations of BT#9 remain effective even in cells with elevated glycolytic activity [67], indicating that mitochondrial targeting may remain effective even in tumors driven by the Warburg effect.

## 5. Conclusions

In conclusion, our study highlights the significant role of the mitochondrial associated-protein MAGMAS in DR PCa cells, particularly its involvement in drug resistance mechanisms. While silencing MAGMAS did not affect baseline cell viability, it markedly enhanced the sensitivity of chemoresistant cells to DTX, partly by disrupting ABCB1, a key drug efflux transporter. Importantly, the sub-therapeutic use of BT#9 to inhibit MAGMAS successfully reversed DTX resistance, suggesting a promising approach for targeting metabolic vulnerabilities in chemoresistant cancer cells. Lastly, PCa primary/local tumor samples with increased RNA expression of MAGMAS, gene alias PAM16, may predict decreased OS. These promising findings not only open new therapeutic possibilities for overcoming chemoresistance but also lay the groundwork for developing personalized treatment strategies aimed at improving survival in advanced PCa.

## Data Availability

Data supporting reported results can be found in a dataset generated during the study. Additional information is available upon request.

## Supplementary Materials

The supporting information can be downloaded at https://www.mdpi.com/article/10.3390/cancers17091535/s1. Figure S1: mmunoblotting analysis of ChromograninA in PCa cell lines. File S1: The original version the Western blot image in Figure 1B; File S2: The original version the Western blot image in Figure 2A; File S3: The original version the Western blot image in Figure 2C.
