# Supplementary material for: Inhibition of Mitochondrial-Associated Protein MAGMAS Resensitizes Chemoresistant Prostate Cancer Cells to Docetaxel
Source: Cancers (Basel). 2025 Apr 30;17(9):1535. doi: 10.3390/cancers17091535 (PMC12072152; doi:10.3390/cancers17091535)
Supplement: Supplementary file 1 [file cancers-17-01535-s001.zip › cancers-3567262-Figure S1.pdf]

Supplementary Figure *S*1

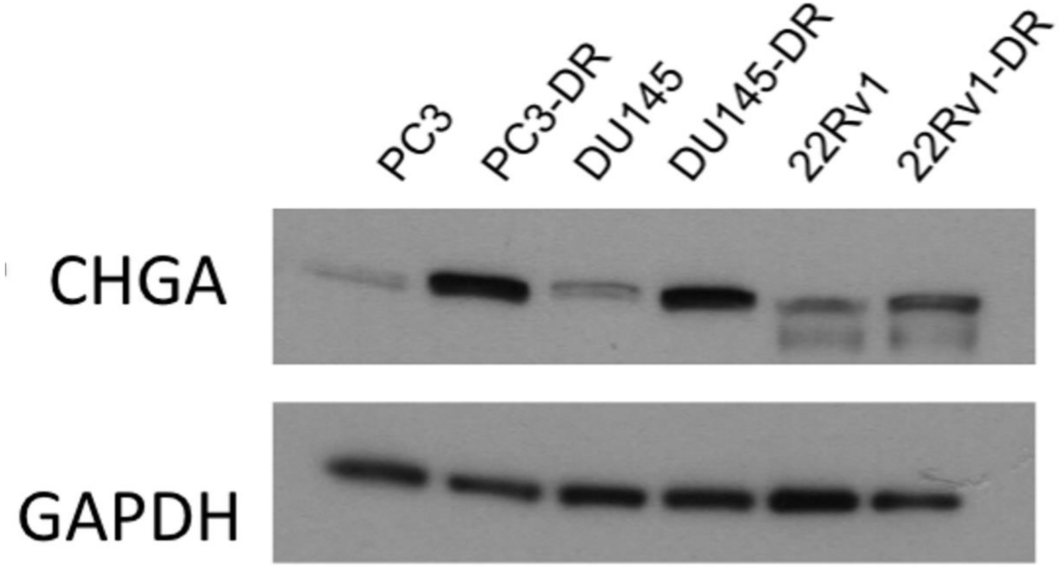

SF1. Immunoblotting analysis of Chromogranin A in PCa cell lines. Representative immunoblots Chromogranin A (CHGA) protein expression in a panel of advanced PCa cell lines. There is increased CHGA expression in DTX-resistant cells (PC3-DR and DU145-DR).
