# Supplementary figures and images for: Inhibition of Mitochondrial-Associated Protein MAGMAS Resensitizes Chemoresistant Prostate Cancer Cells to Docetaxel
Source: Cancers (Basel). 2025 Apr 30;17(9):1535. doi: 10.3390/cancers17091535 (PMC12072152; doi:10.3390/cancers17091535)

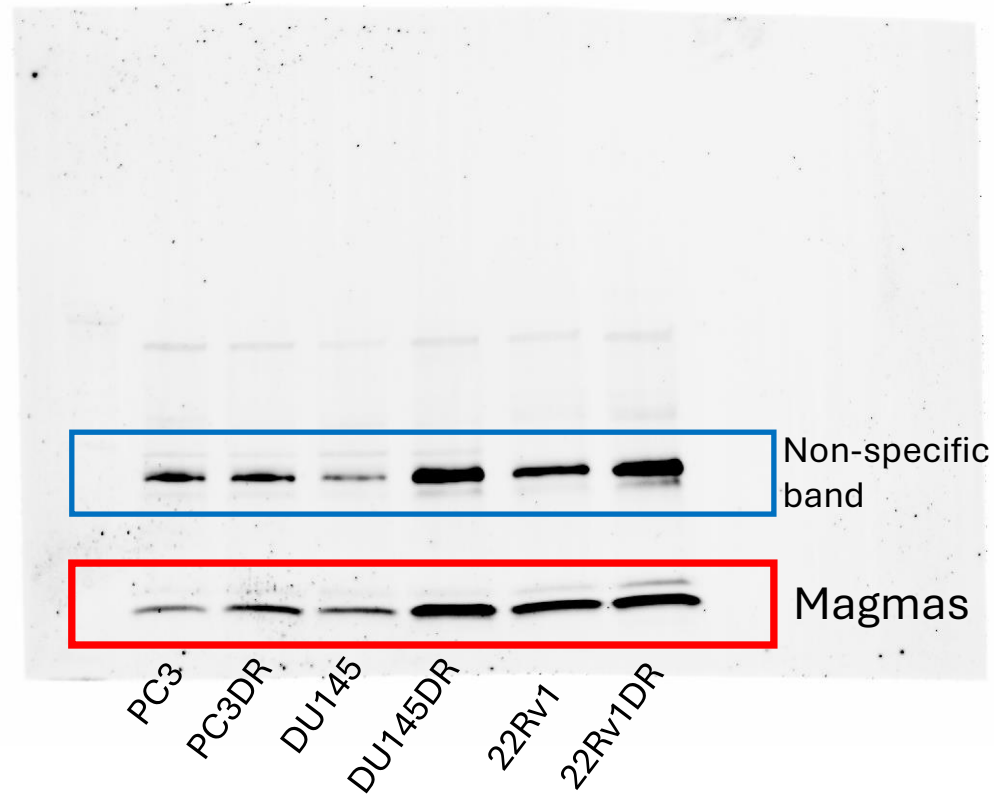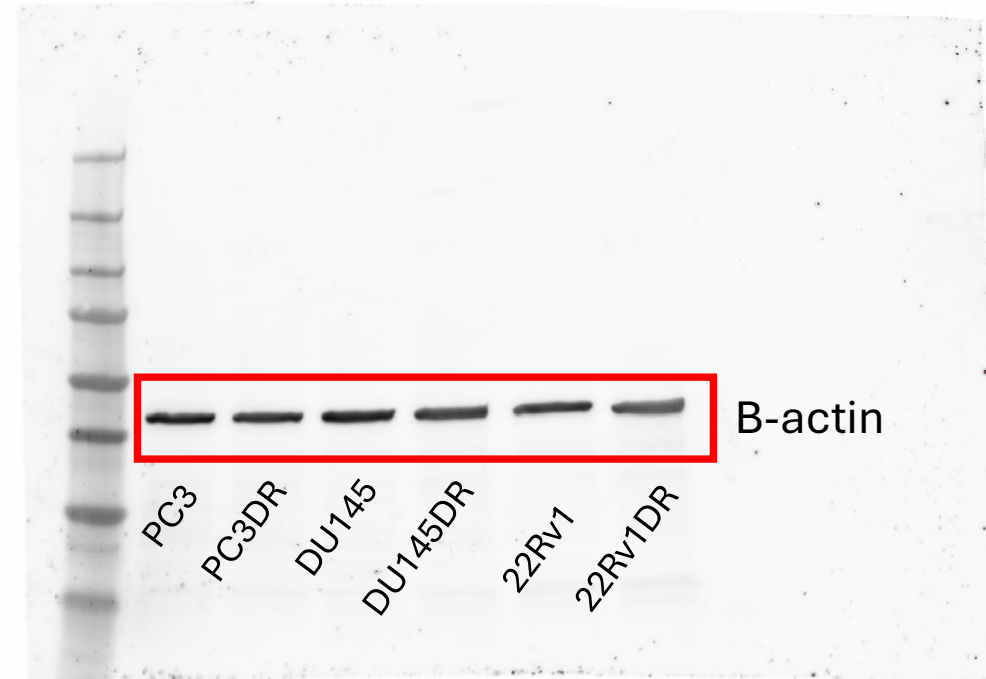

File S1 : The original version the Western blot image in Figure 1B

Supplement: Supplementary file 1 [file cancers-17-01535-s001.zip › cancers-3567262-File S1.pdf]

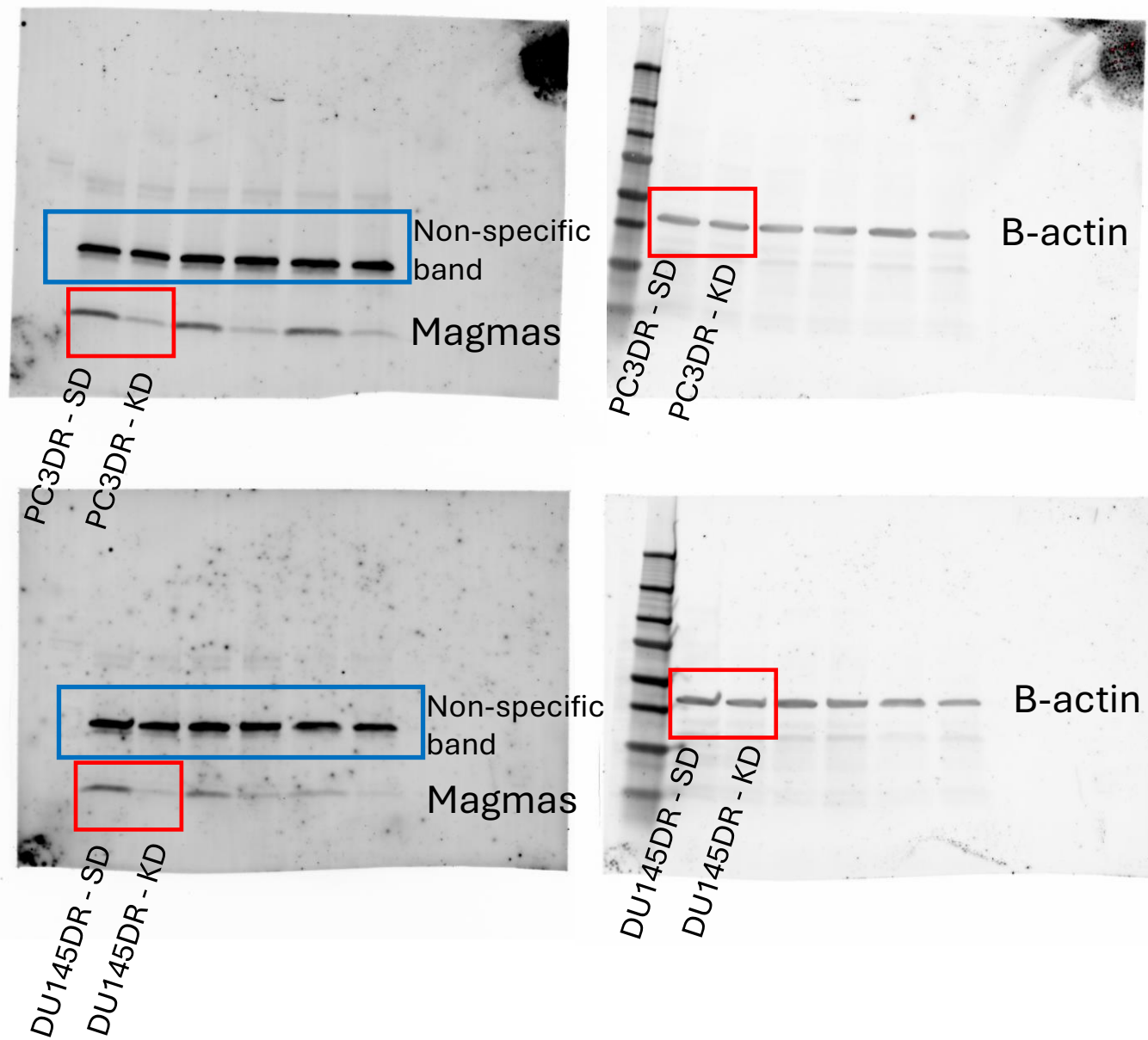

File S2: The original version the Western blot image in Figure 2A

Supplement: Supplementary file 1 [file cancers-17-01535-s001.zip › cancers-3567262-File S2.pdf]

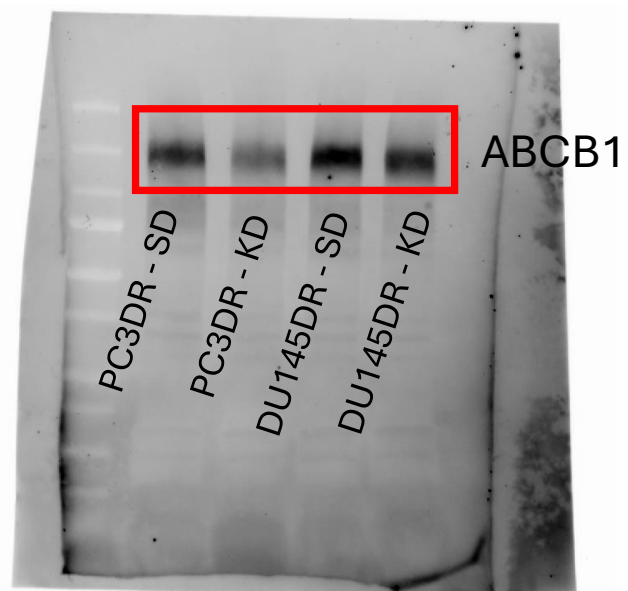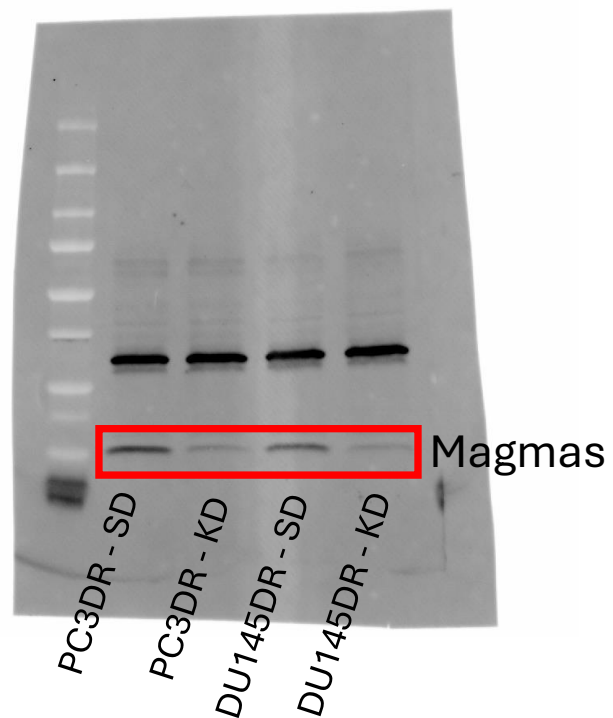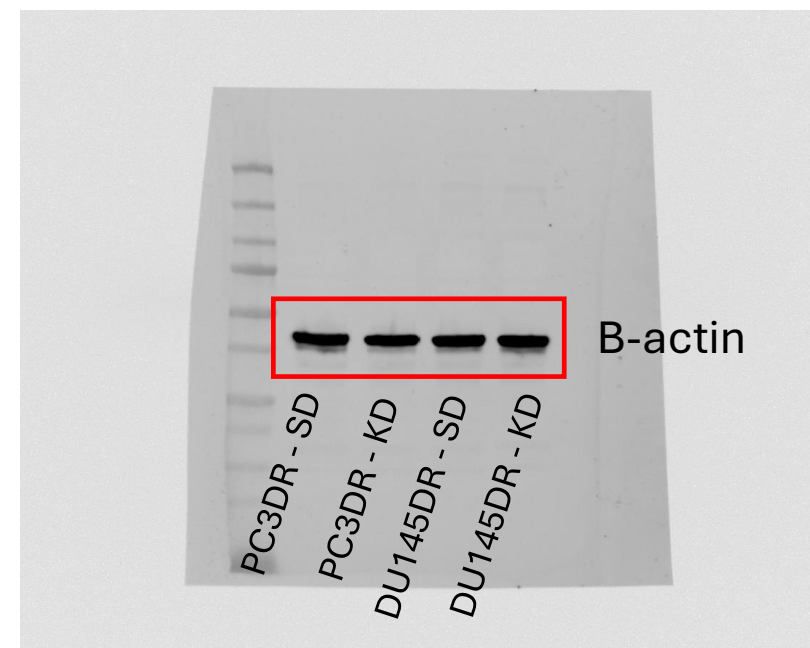

File S3: The original version the Western blot image in Figure 2C

Supplement: Supplementary file 1 [file cancers-17-01535-s001.zip › cancers-3567262-File S3.pdf]
